# Exploring Thrips Preference and Resistance in Flowers, Leaves, and Whole Plants of Ten *Capsicum* Accessions

**DOI:** 10.3390/plants12040825

**Published:** 2023-02-13

**Authors:** Isabella G. S. Visschers, Mirka Macel, Janny L. Peters, Lidiya Sergeeva, Jan Bruin, Nicole M. van Dam

**Affiliations:** 1 Has Green Academy, Spoorstraat 62, 5911 KJ Venlo, The Netherlands; 2 Weerbare Planten, Aeres University of Applied Science, Arboretum West 98, 1325 WB Almere, The Netherlands; 3 Plant Systems Physiology, Radboud Institute for Biological and Environmental Sciences, Radboud University, 6525 AJ Nijmegen, The Netherlands; 4 Laboratory of Plant Physiology, Department of Plant Sciences, Wageningen University, Droevendaalsesteeg 1, 6708 PB Wageningen, The Netherlands; 5 Syngenta, Westeinde 62, 1601 BK Enkhuizen, The Netherlands; 6 Leibniz-Institute for Vegetable and Ornamental Crops (IGZ), Theodor-Echtermeyer-Weg 1, 14979 Großbeeren, Germany; 7 Institute of Biodiversity, Friedrich Schiller University Jena, Dornburger-Str. 159, 07743 Jena, Germany

**Keywords:** *Frankliniella occidentalis*, pollen, nectar, pepper, insect resistance, crop breeding, population development, carbohydrates

## Abstract

*Capsicum* species grown for pepper production suffer severely from thrips damage, urging the identification of natural resistance. Resistance levels are commonly assessed on leaves. However, *Capsicum* plants are flower-bearing during most of the production season, and thrips also feed on pollen and flower tissues. In order to obtain a comprehensive estimate of elements contributing to thrips resistance, flower tissues should be considered as well. Therefore, we assessed resistance to *Frankliniella occidentalis* in flowers, leaves, and whole plants of ten *Capsicum* accessions. Using choice assays, we found that thrips prefer flowers of certain accessions over others. The preference of adult thrips for flowers was positively correlated to trehalose and fructose concentration in anthers as well as to pollen quantity. Resistance measured on leaf discs and thrips population development on whole plants was significantly and positively correlated. Leaf-based resistance thus translates to reduced thrips population development. Results of the flower assays were not significantly correlated with resistance in leaves or on whole plants. This suggests that both leaves and flowers represent a different part of the resistance spectrum and should both be considered for understanding whole plant resistance and the identification of resistant *Capsicum* varieties.

## 1. Introduction

Thrips are widespread insects that cause damage and yield loss in many vegetable crops. They can cause direct damage due to feeding and oviposition as well as indirect damage by the transmission of plant viruses, such as Tospoviruses [1,2,3]. Thrips are polyphagous insects and make use of diverse food sources that are present on the plant. Both leaves [4,5] and the pistil, calyx, petals, and filaments of the flowers can be used for oviposition and feeding [6,7]. Pollen provides an important additional source of nutrients. Both the quantity and quality of pollen can influence thrips behavior and reproductive parameters [8,9,10]. Thrips population growth thus relies on different plant resources that become available during the development of the host plant.

*Frankliniella occidentalis*, also known as Western Flower Thrips, occurs both on leaves and flowers of crop plants. This species was first described in 1895 in California and is now among the most widespread thrips species [11], causing severe yield losses globally. Controlling this and other thrips species is challenging due to the emergence of populations that are resistant to insecticides [12]. Due to the their preference to hide in small narrow places (thigmotactic behavior) [13], insecticides that are still efficient do not always reach their target. Thrips control is therefore mostly achieved by integrated pest management (IPM) [14,15]. Natural crop resistance is one of the elements of successful IPM programs.

*Capsicum* is a genus in the nightshade family, including hot and sweet pepper varieties. In Dutch greenhouse production systems, *Capsicum* plants are severely damaged by *F. occidentalis* [16]. Natural resistance to thrips is a highly desired trait, and several resistant accessions have been identified in this genus [17,18,19,20]. Resistant *Capsicum* accessions can be identified based on feeding or reproduction rates, or on thrips mortality. So far, these resistance parameters have been exclusively assessed on the leaves [21,22,23]. This ignores the fact that thrips also use other resources of the host, such as flowers and pollen. *Capsicum* plants are flower-bearing during several months of the production period. The larvae and adults of *F. occidentalis* are found in high numbers in these flowers [24,25,26]. Most studies using food resources other than leaves focus on the specific role of pollen addition in thrips settling behavior and reproduction [8,9]. For example, the presence of pine pollen significantly influences host leaf selection and settling behavior of *F. occidentalis* [8]. On a poor host (*Arachis hypogaea*, peanut), the addition of pine pollen enhanced reproductive output by 58% [9]. This means that the presence of pollen can enhance attractiveness as well as thrips population growth on flowering plants. In addition, *F. occidentalis* population development was found to be faster on *Rosa chinensis* (Chinese rose) flowers and reached a higher total population size compared with *Gardenia jasminoides* flowers [27]. In tomato, oviposition rates on flowers differed among interspecific hybrid lines [28]. These findings suggest that flowers contribute to overall thrips resistance. To our knowledge, flowers of *Capsicum* accessions have not been screened for thrips resistance yet. Including the screening of flowers and pollen in the search for more resistant varieties is thus essential to obtaining a better view on the differences in thrips resistance among *Capsicum* accessions.

In a previous study, we screened a panel of 40 *Capsicum* accessions for resistance to *F. occidentalis* and *T. tabaci* [20]. Resistance levels were assessed by determining thrips feeding damage on leaf discs. This screening resulted in the selection of 11 *Capsicum* accessions that were classified as resistant or susceptible [29,30]. Determining thrips population development on whole plants of these selected *Capsicum* accessions will provide further insight into whether leaf-based resistance translates to reduced thrips population growth. It remains unclear to what extent the flowers contribute to the differences in thrips resistance among the accessions. Flowers and leaves differ in their morphology, e.g., cuticular wax layers [31,32] and chemical composition [33]. This suggests that leaf and flower resistance levels may differ considerably, and therefore, total plant resistance might not be directly derived from assessing leaf resistance alone. Considering that in commercial production systems the plants will be flowering for most of the production period, the level of resistance in flowers is an important element of total plant resistance to thrips.

Here, we assessed resistance to *F. occidentalis* in flowers, leaves, and whole plants of 10 *Capsicum* accessions and determined whether identified resistance was positively correlated among these plant parts. To this aim, we assessed (1) thrips preference for flowers of the different accessions combined with measurements of pollen quantity and sugar content of the anthers as possible drivers for preference, (2) thrips reproduction on the flowers, and (3) thrips population growth and damage on the whole plants of the 10 selected *Capsicum* accessions [29,30]. We expected that accessions with the most pollen and the highest sugar content were preferred the most and would yield the highest thrips reproduction rates. In addition, we expected that thrips would lose their preference when offered flowers without the pollen. Finally, we expected that the feeding damage on detached leaves and whole plants would be positively correlated with the number of larvae and adult thrips after five weeks.

## 2. Results

### 2.1. Flower Choice Assay

**Figure 1:**
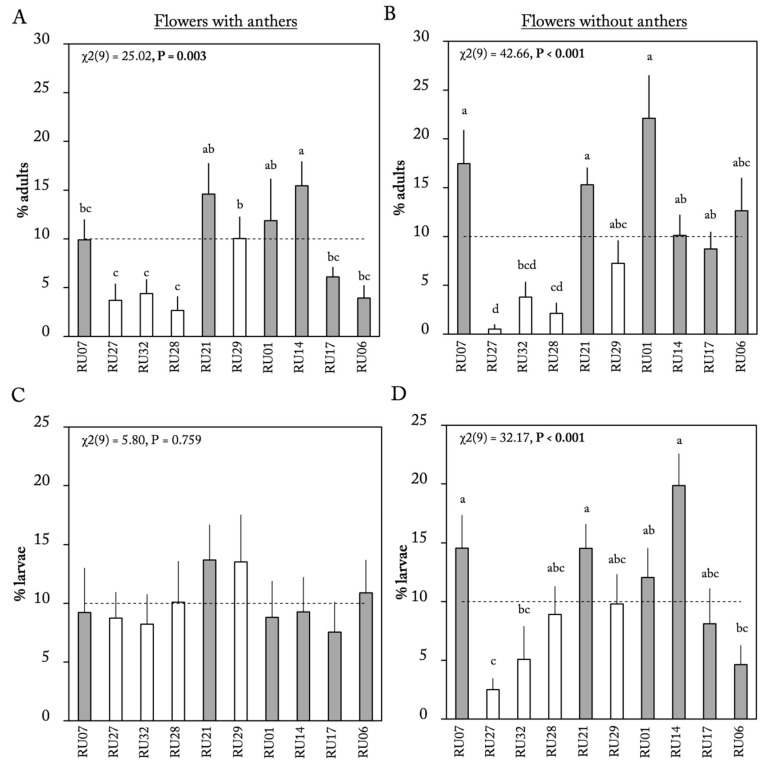
*Frankliniella occidentalis* full choice settling behavior of adults and offspring on flowers with and without anthers of 6 *Capsicum annuum* accessions (gray bars) and 4 *C. chinense* accessions (white bars). (**A**,**B**) Mean (±SE) percentage of adult thrips (*n* = 14–16 replicates per assay) present on flowers with and without anthers after 2 h (*n* = 10 per accession). (**C**,**D**) Mean (±SE) percentage of larvae emerging on each flower with and without anthers after adult thrips were allowed to oviposit for 48 h. Dotted line at 10% in each panel is the distribution under no-choice conditions. *p*-values of overall Friedman ANOVA for dependent data are given in each panel. Different letters indicate a significant difference between accessions (*p* < 0.05, post hoc Wilcoxon signed-rank tests).

In both flower assays, the preference of adult thrips was monitored at three different time points. For most of the accessions, we observed no significant effect of census time point on thrips preference (Figure S1a,b). Differences among accessions were further analyzed using thrips preference after 2 h. Both flower assays showed that thrips preferred to settle on certain accessions over others (Friedman ANOVA, with anthers: χ^2^_(9)_ = 25.02, *p* = 0.003; without anthers: χ^2^_(9)_ = 42.66, *p* < 0.001; Figure 1A,B). Regardless whether anthers were present, the flowers of three *C. chinense* accessions (RU27, RU32, and RU28) were the least preferred (Figure 1A,B, white bars). When anthers were present on the flower, thrips preferred to settle on the flowers of accessions RU21, RU14, and RU01 (15.4%, 14.6%, and 11.8%, respectively, Figure 1A). When anthers were removed, most thrips were found on accessions RU07, RU21, and RU01 (17.5%, 15.3%, and 22.1%, respectively, Figure 1B). We observed no significant differences in the percentage of larvae emerging from the flowers of different accessions when flowers had anthers (Friedman ANOVA, χ^2^_(9)_ = 5.8, *p* = 0.759, Figure 1C). However, when anthers were removed at the time the flowers were exposed to adults, the percentage of emerged larvae differed among the accessions (Friedman ANOVA, χ^2^_(9)_ = 32.17, *p* < 0.001, Figure 1D). In flowers without anthers, most larvae emerged from the flowers of accessions RU07, RU21, and RU14 (14.5%, 14.5%, and 19.8%, respectively) while the lowest percentage of larvae emerged from the flowers of accession RU27 (2.5%, Figure 1D). The percentage of larvae emerging from flowers was not correlated with adult preference in either flower assay (Spearman rank correlation, with anthers: *p* = 0.448, ρ(9) = 0.27; without anthers: *p* = 0.178, ρ(9) = 0.47; Table 1).

**Figure 2:**
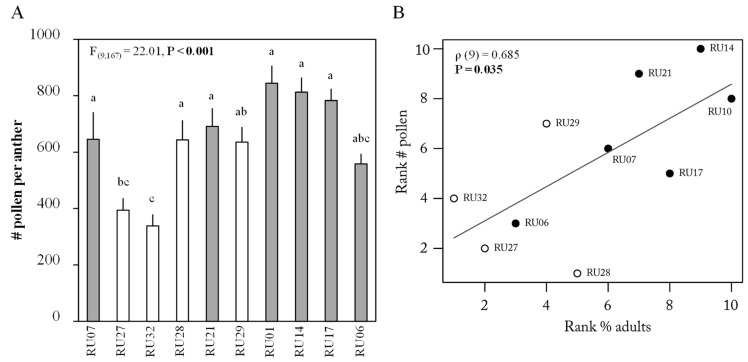
(**A**) Mean (±SE) number of number (#) of pollen grains per anther of 6 *Capsicum annuum* accessions (gray bars) and 4 *C. chinense* accessions (white bars) (*n* = 14–21 per accession). *p*-value of overall ANOVA for independent data is given in the panel. Different letters indicate a significant difference between accessions (*p* < 0.05, post hoc Tukey HSD). (**B**) Rank correlation between the number (#) of pollen grains per anther and number of adult thrips observed on the flowers. *p*-values and rho (ρ) of Spearman correlation are given in the graph. Black dots represent *C. annuum* accessions; white dots represent *C. chinense* accessions.

**Figure 3:**
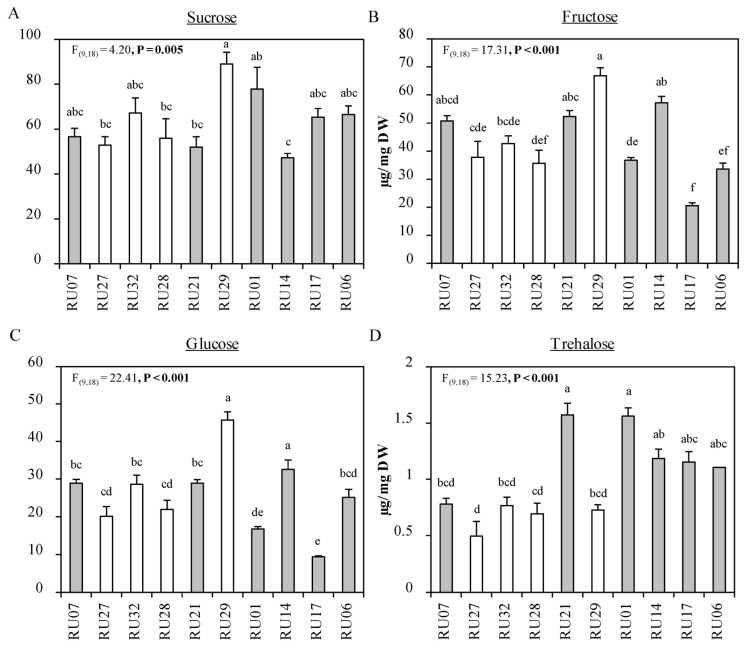
Mean (±SE) concentration of four different carbohydrates: sucrose (**A**), fructose (**B**), glucose (**C**), and trehalose (**D**) in anthers of 6 *Capsicum annuum* accessions (gray bars) and 4 *Capsicum chinense* accessions (white bars) (*n* = 2–3). *p*-value of overall ANOVA for independent data is given in each panel. Different letters indicate a significant difference between accessions (*p* < 0.05, post hoc Tukey HSD).

Pollen quantity per anther significantly differed among the accessions (ANOVA, F_(1,167)_ = 22.01, *p* < 0.001, Figure 2). The lowest numbers of pollen grains per anther were observed in the *C. chinense* accessions RU27 and RU32 (Figure 2). Next, we tested whether differences in adult preference were linked to pollen quantity. Correlation analysis showed that there was a significant positive correlation between preference and number of pollen grains (Spearman correlation, *p* = 0.035, ρ(9) = 0.685, Figure 2B); adult thrips were more likely to settle on plants carrying more pollen grains. Finally, the major carbohydrate in the anthers was sucrose (47% of total carbohydrates detected), followed by fructose (32%), glucose (19%), and trehalose (0.7%) (Figure 3). The concentration of all four carbohydrates differed significantly among the accessions (Sucrose: F_(9,18)_ = 4.20, *p* = 0.005, Fructose: F_(9,18)_ = 17.31, *p* < 0.001, Glucose: F_(9,18)_ = 22.41, *p* < 0.001, and Trehalose: F_(9,18)_ = 15.23, *p* < 0.001 Figure 3). Accession RU29 contained the highest absolute content of glucose, fructose, and sucrose (45.7, 66.9, and 89 µg/mg DW, respectively, Figure 3). No significant correlation between sugar concentration and percentage of thrips on flowers with anthers could be detected for glucose and sucrose (glucose: ρ = 0.503, *p* = 0.143 and sucrose: ρ = −1.11, *p* = 0.759). A positive significant correlation between these two parameters could be detected for fructose and trehalose (ρ = 0.624, *p* = 0.06 and ρ = 0.77, *p* = 0.014, respectively).

### 2.2. Leaf Disc Choice Assay

**Figure 4:**
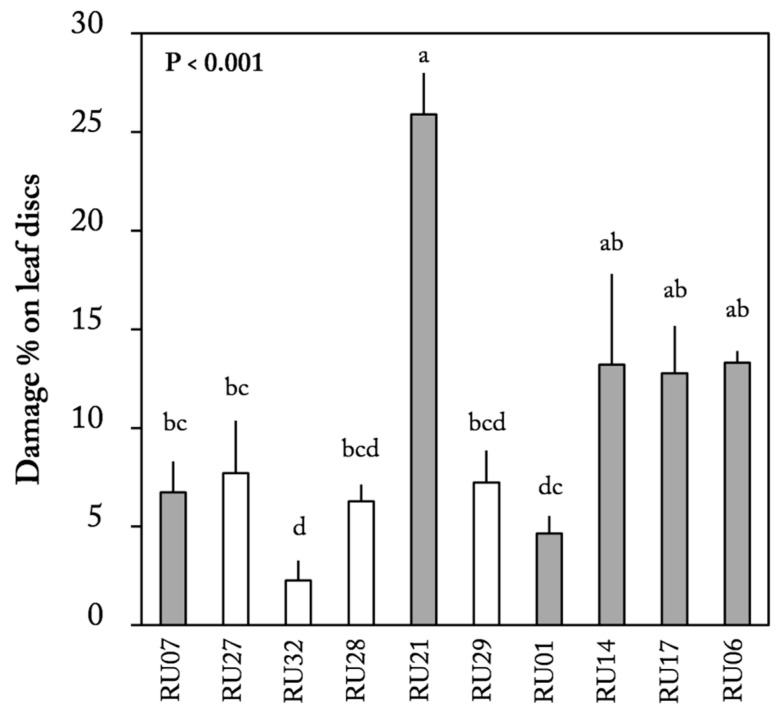
*Frankliniella occidentalis* choice assay on leaf discs of 6 *Capsicum annuum* accessions (gray bars) and 4 *C. chinense* accessions (white bars). Mean (±SE) damage percentage of leaf discs (*n* = 9 per accession). *p*-values of overall main effects of accession on damage percentage (Friedman ANOVA for dependent data) are given in the graph. Different letters indicate significant differences between accessions (*p* < 0.05, post hoc Wilcoxon signed-rank tests).

A leaf disc choice assay was conducted using the same plants as in the flower assays. There was a significant difference in damage on leaf discs among accessions (Friedman ANOVA, χ^2^_(9)_ = 39.23, *p* < 0.001, Figure 4). The leaf discs of accession RU21 were damaged the most and the leaf discs of accession RU32 the least (25.9% and 2.3% damage, respectively, Figure 4). Damage on leaf discs was not correlated with preference, neither for flowers with nor for flowers without anthers (Spearman rank correlation, with anthers: *p* = 0.427, ρ(9) = 0.28; without anthers: *p* = 0.973, ρ(9) = −0.018; Table 1).

### 2.3. Thrips Population Development

**Figure 5:**
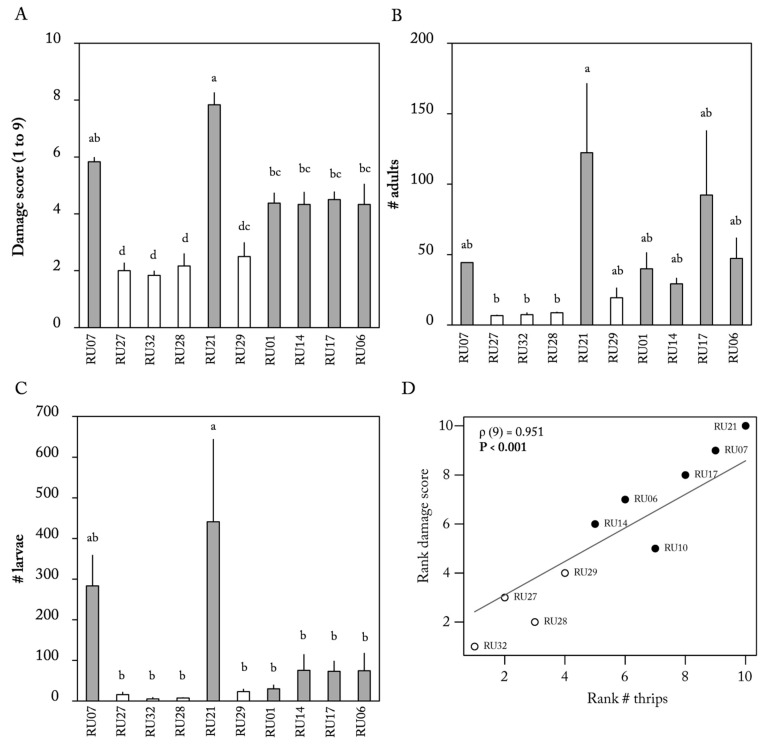
*Frankliniella occidentalis* population development on 6 *Capsicum annuum* accessions (gray bars) and 4 *C. chinense* accessions (white bars) 5 weeks after plants were inoculated with 50 thrips. (**A**) Mean (±SE) *F. occidentalis* damage scores, (**B**) number (#) of larvae, and (**C**) number (#) of adults (*n* = 3–4 per accession). Different letters indicate a significant difference between accessions (*p* < 0.05, post hoc Tukey HSD). (**D**) Rank correlation between damage score and total number of thrips. *p*-values and rho (ρ) of Spearman correlation are given in the graph. Black dots represent *C. annuum* accessions; white dots represent *C. chinense* accessions.

Thrips population development was monitored on the same 10 *Capsicum* accessions over a period of five weeks. We observed a significant effect of accession and time point on damage scores and number of larvae (ANOVA, damage score: accession effect F_(9,61)_ = 51.918, *p* < 0.001 and time point effect F_(2,61)_ = 6.22, *p* < 0.001; number of larvae: accession effect F_(9,62)_ = 9, *p* < 0.001 and time point effect F_(2,62)_ = 5.31, *p* < 0.007; Figure S2). For adults, we only observed a significant effect of accession but not of time point (number of adults: accession effect F_(9,62)_ = 6.29, *p* < 0.001 and time point effect F_(9,62)_ = 1.65, *p* = 0.200, Figure S2). Accession RU21, which had the greatest thrips damage also supported the highest numbers of adults and larvae (Figure 5A–C). All *C. chinense* accessions (RU27, RU32, RU29, and RU28) showed the least thrips damage (score: 2.0, 1.8, 2.1, Figure 5A) and supported low numbers of both larvae and adult thrips (Figure 5B,C). Correlation analysis confirmed a strong correlation between thrips damage observed and the total number of thrips present on these same plants (Spearman correlation, *p* < 0.001, ρ (9) = 0.95, Figure 5D). Percentage damage on leaf discs and the total thrips population were significantly and positively correlated (Spearman correlation, *p* = 0.039, ρ (9) = 0.67, Table 1). The results of the flower assays were not significantly correlated with any of the thrips population development parameters (Table 1).

## 3. Discussion

In our study we showed that thrips prefer flowers of certain *Capsicum* accessions over others. Three *C. chinense* accessions were the least preferred, regardless of whether or not anthers were present on the flowers. The preference of adult thrips for flowers with anthers was correlated to absolute trehalose and fructose content and to pollen quantity in complete flowers. Without anthers, thrips still preferred some accessions over others, although preference was different compared with flowers with anthers. This suggests that factors other than pollen also drive preference. The percentage of thrips offspring emerging from flowers differed significantly among accessions when thrips were offered flowers without anthers. The results of our flower assays did not correlate with thrips preference in the leaf discs choice assays or thrips performance in the whole plant no-choice experiments. Our earlier studies already showed that leaves of vegetative and flowering *Capsicum* accessions were not equally resistant [20,29]. Our current findings show that resistance to thrips may also differ between plant organs.

Our data suggest that pollen quantity is a possible driver for preference in intact flowers. High numbers of pollen grains per anther were positively correlated with the presence of adult thrips. Our results complement previous studies on the positive role of pollen in settling behavior of *F. occidentalis* [8,9]. The addition of pine pollen (*Pinus elliottii*) on tomato (*Lycopersicon esculentum*) leaflets significantly increased preference compared with leaflets without the addition of pollen. Significant difference in preference was lost after several days, indicating that the pollen provided a food source that was depleted after several days [8] and supporting our findings on the role of pollen quantity as driver for preference.

In flowers without anthers, the settling behavior was still significantly different among the accessions, but changed compared with the choice assay with anthers. This suggests that additional factors play a role in preference of thrips for *Capsicum* flowers, which are overruled when pollen grains are present. Possibly, differences in the quantity or quality of floral nectar among the accessions could be such a factor. Nectar contains primary metabolites, such as sugars and amino acids, which provide a food source for pollinators and thrips alike. On the other hand, floral nectar can contain toxic or repellent secondary metabolites, which affect for example honeybees, sunbirds, and several floral visitors, as was shown for *Nicotiana attenuata* [34,35,36]. Finally, volatiles emitted from the floral tissues can attract or repel insects. For example, the chemical analysis of the floral scent compounds of *Ligustrum japonicum* resulted in the identification of phenylacetaldehyde that stimulates flower visiting by *Pieris rapae* [37], while decalactone in *Osmanthus fragrans* discourages the foraging behavior of *P. rapae* [38]. Similarly, *Thrips hawaiiensis* shows a preference for four different flowers in a set of 21 flowering host plant species, which is driven by olfactory ques [39]. Since our experiments were conducted in closed Petri dishes, volatiles emitted from the flowers of the different accession might have mixed reducing the effect in our experiment compared with an open setup. However, based on research in other plant–insect systems, we still expect differences in volatile profiles to contribute to thrips preference. Volatile profiles and differences in floral nectar composition among accessions have not yet been studied in *Capsicum* flowers, but they might be of importance in understanding the preference of adult thrips for specific flowers.

Adult thrips preference was positively correlated with the concentration of trehalose and fructose in anthers, but not to sucrose and glucose content. Sugars are an important feeding stimulant for thrips and other insects [40,41]. Differences in sugar concentrations in leaves were related to susceptibility in several plant species. For example, high levels of sugars were linked to high levels of *Thrips tabaci* feeding damage on *Peumus boldus* leaves [42], onion (*Allium cepa*), and leek (*Allium ampeloprasum*) [43,44]. In *Capsicum* leaves, high levels of sucrose were related to susceptibility to *F. occidentalis* [29]. In addition to these above studies on leaf sugar contents, our data show that high levels of fructose and trehalose, but not sucrose, in the anthers are linked to thrips preference in flowers. Trehalose is a disaccharide that is known among others as the “insects’ blood-sugar” and is present in high concentrations in the hemolymph in insects where it serves as source of energy and carbon [45]. In insects, it plays, among other things, an important role in the development and flight of insects [46]. In plants, it is involved in the response to a range of abiotic and biotic stresses, where it provides direct resistance to insects or functions as a signaling molecule [47,48]. In *Arabidopsis* and tomato, trehalose negatively affects the survival of the of green peach aphid (*Myzus persicae*) nymphs, thereby reducing the severity of infestation [48,49,50]. In contrast to these reports, our results suggest that trehalose and fructose in anthers may enhance thrips infestation on *Capsicum* flowers, although the underlying mechanism of this preference and performance is not known.

Research on the response of *F. occidentalis* to color has been done, mostly on sticky trap efficiency for monitoring thrips infestation in green houses. Several of these studies have been done on white, yellow and blue sticky plates. Blue traps were found to be the most attractive for thrips [51]. Interestingly, a study by Van Tol, et al. [52] showed that preference for blue versus yellow sticky traps changed depending on the surface structure, in this case the glaze of the glue that was used. A study on chrysanthemums showed significant differences in the number of adult thrips in flowers based on color (Rogge and Meyhöfer 2021). Similar observations have been found on roses [53]. Overall, this suggest that a combination of color and surface structure might contribute to thrips preference for a specific color. The flowers of the accessions that were used in our experiments were all slightly yellow in color. However, reflectiveness of the surface of flower parts could have differed among the accessions. This might have resulted in differences in reflection spectra that are not perceived by the human eye, but are perceived by the thrips. Hyperspectral imaging is a non-invasive method for plant phenotyping that uses color reflecting information over a large range of the light spectrum (Lowe et al. 2017). Analyses of hyperspectral data of the flowers used in our experiment might provide subtle differences in reflection spectra that contribute to preference of thrips for flowers of certain accessions over others. 

We further showed that the thrips reproductive output on flowers differed among the accessions. Interestingly, this effect was only observed when anthers were absent. Our data could not reveal whether this was due to differences in oviposition or egg mortality rates among the accessions. Our previous studies, using leaf discs, showed that the composition of the cuticular wax layer may play a role in thrips preference in *Capsicum* [54]. Moreover, secondary metabolites such as monomer and dimer acyclic diterpene glycosides (capsianosides) are linked to thrips feeding resistance in *Capsicum* leaves [29,55]. We do not know whether the flowers also contain these diterpene glycosides, but they are also found in the *Capsicum* fruits [56]. The presence of additional high-quality food sources, such as anthers and pollen, may have alleviated the negative effect of defensive compounds in flowers because *C. chinense* flowers were even less preferred in assays without anthers in the flowers. Altogether, our results indicate that preference and reproductive output of *F. occidentalis* in flowers is governed by a combination of pollen quantity, and trehalose and fructose concentration in anthers with possible olfactory ques, nectar composition, and secondary defense compounds in flower tissues.

To obtain a complete overview of resistance to thrips in these *Capsicum* accessions, leaf-based resistance and whole plant thrips population development were assessed as well. Substantial consistent differences in thrips damage and population size were observed between the different accessions in both the leaf assays and the whole plant assays even though light and temperature conditions were slightly different among these experiments. Accession RU32 was identified as consistently resistant, evidenced by low damage levels and small thrips populations. These findings complement our previous work on thrips resistance in this accession. RU32 was also identified as resistant in leaf disc assays of plants in the flowering stage by Macel, Visschers, Peters, Kappers, De Vos and van Dam [29]. Finally, our data showed a positive correlation between leaf damage on leaf discs taken from plants in the reproductive stage and thrips population size. Measuring thrips leaf damage in *Capsicum* using a high-throughput leaf disc assay thus provides as a good indication of thrips infestation and whole plant resistance. However, flower resistance and resistance in detached leaves and whole plants were not correlated in our study, meaning that each organ may contribute uniquely to whole-plant thrips resistance. This suggests that exploring resistance levels and underlying mechanisms of resistance to thrips in generative plant parts, in addition to leaf-based resistance, is of importance for identifying robust resistant accessions for breeding.

## 4. Material and Methods

### 4.1. Plant Material

We used two *Capsicum* species, *C. annuum* and *C. chinense* (Table 2). Original seeds were obtained from the Centre for Genetic Resources (CGN), Wageningen University and Research Centre, The Netherlands (http://cgngenis.wur.nl/ (January 2015)) and from Syngenta (Enkhuizen, The Netherlands). An F2 (self-pollinated) population was produced at the Radboud University (Nijmegen, The Netherlands) using six plants per accession.

### 4.2. Plant Rearing

Seeds for the flowering and leaf disc assay were germinated in closed plastic cups (Ø 7 cm) on sterile glass beads (Ø 1 mm) in a climate cabinet (Snijders Labs, Tilburg, The Netherlands) at L16:D8 light–dark regime and temperature set to 30 °C/20 °C (day/night). When the first two true leaves had developed, the seedlings were transplanted to pots (11 cm × 11 cm × 12 cm) containing potting soil (Potting soil 4, Horticoop, Bleiswijk, The Netherlands). The pots were placed on tables in a greenhouse, inside an insect-free net cage (Rovero 0.30 mm gauze, 7.50 m × 3 m × 2.75 m) at 16 h photoperiod and minimum temperatures set to 20 °C/17 °C (day/night). Natural light was supplemented with Greenpower lights (400 V/1000 W, Phillips, Amsterdam, The Netherlands) when below 200 Watt m^−2^. Plants were inoculated with a biological control agent, *Amblyseius swirskii* (Koppert Biological Systems, Berkel en Rodenrijs, The Netherlands) every 4 to 6 weeks as prevention for unwanted thrips infestation.

Population development assays on whole plants were conducted in a greenhouse with windows sealed with gauze in Enkhuizen, The Netherlands (52°42′3.676″ N, 5°6′12.243″ E). Temperatures were set to 24 °C continuous and light was supplemented when below 400 W/m² using 10.000 lux Son-T lamps. Seeds were germinated in sowing trays with fine peat soil; after two weeks, seedlings were transplanted to pots (7 × 7 × 8 cm) and placed in the greenhouse.

### 4.3. Insect Culture

*Frankliniella occidentalis* culture conditions were the same as described by (Visschers et al. 2018a). The leaf disc assay was performed using synchronized L1/L2 larvae that were starved for 24 h prior to experiments. Flower experiments were conducted with adult thrips. Testing conditions and thrips colony rearing conditions were kept constant during the experimental period. Thrips used for the population development assay were reared on common bean plants (*Phaseolus vulgaris*).

### 4.4. Full Choice Settling Behavior Assay on Flowers

Two flower experiments were conducted using: (1) flowers with dehiscent anthers and (2) flowers without anthers. For the first experiment, fresh flowers with recently opened anthers were collected from the plants. Flowers were collected from three to five plants per accession. Flowers were pooled together per accession in a 50 mL tube and transported to the laboratory for further experiments. In the laboratory, one flower from each accession was placed in a Petri dish (Ø 150 mm) with the pedicel in a drop of slightly liquid sterile agar, thereby forming a circle of flowers. Each Petri dish (*n* = 10) contained 10 flowers, each representing one of the ten accessions. All flowers were placed at equal distance from the center of the Petri dish with the anthers facing the center. Thereafter, 14–16 adult thrips were randomly chosen from the culture. They were placed in the center of the Petri dish using a paint brush, and the Petri dishes were closed with Parafilm. The number of thrips present on each flower in the Petri dish was monitored at 10 min, 1 h, and 2 h after closing the Petri dishes.

For the second experiment, flowers with the anthers still closed were collected. Anthers were removed with a tweezer before conducting the experiment to prevent opening of anthers during the experimental period. The experimental setup was the same as described above.

After 48 h, the thrips were removed, and the flowers were separated into individual Petri dishes (Ø 55 mm). A 1 cm piece of common bean (*Phaseolus vulgaris* L.) was placed next to the flower in the Petri dish. A 1 cm^2^ piece of filtration paper was placed at the bottom to prevent condensation droplets from forming due to an excessive buildup of humidity. The filtration paper was changed after 48 h. The total number of emerging larvae from each flower was determined after 10 days. Since the females insert the eggs into the flower tissues, the number of eggs in the flowers could not be determined, not even after staining.

### 4.5. Pollen Counting

Flowers with closed anthers were collected from each accession for determining pollen quantities (*n* = 14–21 flowers per accession). Flowers were collected from three to five plants per accession and pooled. Pollen numbers per flower measure were based on impedance flow cytometry (AMPHA Z30, Amphasys AG, Lucerne, Switzerland; Heidmann and Di Berardino [57]). The anthers from each flower were cut into four transversal sections and vortexed in an Eppendorf to release the pollen. One mL of AmphaFluid6 was added and poured over a filter (50 µm). An aliquot of 500 µL solution was then measured at 12 MHz using the following settings: Level = 0.04, Modulation = 4, Amplification = 6, Demodulation = 2, and Pump = 60 to determine the pollen numbers.

### 4.6. Soluble Carbohydrates in Anthers

Anthers were collected from two to three plants per accession and frozen in liquid nitrogen before freeze drying overnight (*n* = 2–3). Soluble carbohydrates were determined according to [58], with some modifications. Six milligrams of freeze-dried and ground anthers were transferred to a 2 mL Eppendorf tube and homogenized in 1 mL of methanol (80% *v*/*v*) with the addition of melezitose (400 mg/L) as the internal standard. Samples were incubated in a water bath for 15 min at 76 °C. After extraction, samples were evaporated during 2–2.5 h till dryness under vacuum (SpeedVac Concentrator, Savant SC210A and refrigerated vapour trap, Savant RVT5105, Thermo, Waltham, MA, USA). The extract was suspended in 0.5 mL of milliQ water, thoroughly vortexed, and then centrifuged for 5 min at 17,000× *g* in an Eppendorf centrifuge. The supernatant was diluted 5-fold in milliQ water and injected into a Dionex HPLC system (Dionex, Sunnyvale, CA, USA) to analyze soluble carbohydrates, using a CarboPacTM PA1, 4 × 250 mm analytical column (Dionex) preceded by a CarboPacTM PA1, 4 × 50 mm guard column, a gradient pump module (ICS-5000+ DP, Dionex), and an ED40-pulsed electrochemical detector (Dionex). Mono-, di-, and trisaccharides were separated by elution in an increasing concentration of NaOH (20–85 mM) with a flow rate of 1 mL per minute. Peaks were identified by co-elution of standards. Sugar quantity was corrected by means of the internal standard and expressed in micrograms of sugar per milligram of dry material.

### 4.7. Leaf Disc Assay

Leaf samples of the same plants as used for harvesting the flowers were obtained from the apical part of the plants in the flowering stage. The leaf disc choice assay was conducted using the methods described by [20]. Leaf discs (1.5 cm Ø) were punched from leaves using a cork borer, thereby avoiding the mid-vein. A leaf disc from each accession was placed on a drop of 1.5% slightly liquid agar with the abaxial side up in a Petri dish (9 cm Ø). Each Petri dish (*n* = 10) thus contained 10 leaf discs (placed in a circle), each representing 1 of the 10 accessions. Nine Petri dishes were inoculated with thrips. Per inoculated Petri dish, 22 *F. occidentalis* larvae were placed in the middle of the dish. All Petri dishes were sealed with Parafilm and placed in a climate cabinet (Economic Delux 432 L with TL lights; Snijders Labs, Tilburg, The Netherlands) at 25 °C and an L16:D8 photoperiod. Petri dishes without thrips were directly sealed with Parafilm and used for area correction during image analysis. After 48 h, leaf discs were analyzed, and thrips feeding damage was determined, including all discoloration of the leaf disc caused by thrips feeding. Image processing and quantification of feeding damage in mm^2^ was performed using ImageJ Fiji (version 2.0.0 with Java 1.6.0_24) [59] and Ilastik (version 1.13) [60] according to the protocol described by Visschers, et al. [61]. Ilastik was trained using four to six leaf discs per accession.

### 4.8. Population Development on Whole Plants

Non-flowering plants of each accession (*n* = 3–4 plants per accession and time point) were placed in an individual thrips-proof gauze cage and inoculated with 50 adult (female and male) thrips, 26 days after sowing. Cages were closed using plastic binding strips. The thrips population on each individual plant was collected 2, 3, and 5 weeks later. Thrips collection was done by opening the cage and carefully lifting the plants out of the cage. The plants were rated for damage using a relative scale from 9 (susceptible, very heavy silvering, large part of the leaf damaged, leaf drop, and heavy growth deformation of young leaves) to 1 (resistant, no silvering damage, and no leaf deformation) and immediately thereafter were immersed in 70% ethanol. The plants were thoroughly mixed in the solution to remove the thrips. The ethanol containing the thrips was filtered, and the total numbers of adults and larvae were then counted using a microscope. All of the accessions contained one to a maximum of three plants (out of three to four plants in total) that were flowering after five weeks, except for accessions RU28 and RU29.

## 5. Statistical Analysis

All statistical analyses were performed using R Version 1.0.153 (R Core Team, Vienna, Austria; 2016).

### 5.1. Choice Assay Flowers

Adult thrips preference for flowers of each accession was calculated as the relative number (%) of thrips per flower in a Petri dish (i.e., number of thrips per flower/total number of thrips per Petri dish × 100). Data were analyzed using the non-parametric Kruskall–Wallis test to analyze the effect of time point within an accession. Differences among accessions of data 2 h after inoculation of the Petri dish were analyzed using the Friedman ANOVA for dependent data; when a significant effect was detected, a post hoc Wilcoxon signed-rank test with FDR correction was conducted.

The relative number of larvae (%) per flower in a Petri dish was determined by % of larvae per flower/total number of larvae per Petri dish × 100. Data were analyzed using the same methods as described for the thrips preference data.

To enable comparison of adult thrips preference and the total pollen numbers, each parameter was transformed to ranks from 1 to 10. The accession with the lowest score (average lowest pollen numbers or lowest percentage of thrips) received a rank of 1, while the accessions with the highest score (average highest pollen numbers or highest percentage of thrips) received a rank of 10.

### 5.2. Choice Assay Leaf Discs

Thrips choice assay data were standardized as relative amount eaten (%) per leaf disc of the total amount eaten in a Petri dish. Data were then analyzed with a Friedman ANOVA for dependent data. Post hoc pairwise differences in thrips damage between accessions were analyzed with a Wilcoxon signed-rank test with FDR correction for multiple comparisons. Significant effects were always reported with alpha set to 0.05.

### 5.3. Population Development

Data of thrips population size were analyzed using two-way ANOVA to assess the effects of time point and accessions on damage scores, the numbers of larvae, and the numbers of adult thrips. Post hoc pairwise differences between accessions were analyzed with Tukey HSD, with data obtained five weeks after plants were inoculated with thrips.

To enable comparison of the total number of thrips (adults + larvae) and damage scores, each parameter was transformed to ranks from 1 to 10. The accession with the lowest score (average lowest number of thrips or lowest damage score) received a rank of 1, while the accessions with the highest score (average highest number of thrips or highest damage score) received a rank of 10.

### 5.4. Pollen and Anther Data

Pollen numbers and absolute sugar content data were analyzed using one-way ANOVA for independent data, and post hoc pairwise differences between accessions were analyzed with Tukey HSD.

### 5.5. Correlation Analyses between Parameters

All correlation analyses between parameters and experiments were performed using Spearman rank correlations.

## Figures and Tables

**Table 1:** Spearman rank correlation matrix between parameters of the leaf disc assay, the flower assay, and the population development experiment. The “# thrips” represents number of thrips in the population development experiments. “Anthers +” represents experiments with anthers, while–represents experiments without anthers. Bold represents significant correlations (*p* < 0.05).

				*p*-Values						
					Flower Assays					
					Adults		Offspring		Population Development	
				Leaf Disc Assay	Anthers +	Anthers -	Anthers +	Anthers -	Damage Score	# Thrips
**ρ**			**Leaf disc assay**		0.427	0.973	0.178	0.811	0.166	0.039
	**Flower assay**	Adults	Anthers +	0.284		0.143	0.448	**0.008**	0.113	0.133
			Anthers -	−0.018	0.503		0.657	0.178	0.104	0.178
		Offspring	Anthers +	0.467	0.273	0.163		0.33	0.47	0.427
			Anthers -	0.091	**0.806**	0.467	0.345		0.104	0.166
		**Population development**	Score	0.479	0.539	0.551	0.261	0.551		**<0.001**
			# Thrips	**0.673**	0.515	0.467	0.284	0.479	**0.951**	

**Table 2:** Overview of the 10 *Capsicum* accessions used in this research.

Species	Accession Code	Ru Code	Accession Name	Geographic Origin
*C. annuum*	CGN16913	RU07	Liebesapfel	Germany
*C. chinense*	CGN16994	RU27	RU 72–194	Brazil
*C. chinense*	CGN16995	RU32	RU 72–241	Brazil
*C. chinense*	CGN17004	RU28	No.1736; PI 281428	Suriname
*C. annuum*	CGN17227	RU21	Bodroghalmi	Hungary
*C. chinense*	CGN21557	RU29	No.4661; PI 159236	United States of America
*C. annuum*	CGN22151	RU10	Ta Pien Chiao; PI 162607	China
*C. annuum*	CGN23222	RU14	Keystone Resistant Giant	United States of America
*C. annuum*	CGN23289	RU17	Long Sweet	Zambia
*C. annuum*	CGN23765	RU06	CM 331; Criollos de Morelos	Mexico

## Data Availability

The datasets generated during and/or analyzed during the current study are available from the corresponding author upon reasonable request.

## Supplementary Materials

The following supporting information can be downloaded at: https://www.mdpi.com/article/10.3390/plants12040825/s1, Figure S1: *Frankliniella occidentalis* full choice behavior assay on flowers with and without anthers after 10 minutes (white bars), 1h (grey bars) and 2h (black bars) of 10 *Capsicum* accessions. Figure S2: *Frankliniella occidentalis* population development on 10 *Capsicum* accessions 2 (white bars), 3 (grey bars) and 5 weeks (black bars) after inoculation.
