# Supplementary material for: Exploring Thrips Preference and Resistance in Flowers, Leaves, and Whole Plants of Ten Capsicum Accessions
Source: Plants (Basel). 2023 Feb 13;12(4):825. doi: 10.3390/plants12040825 (PMC9960883; doi:10.3390/plants12040825)
Supplement: Supplementary file 1 [file plants-12-00825-s001.zip › plants-2160966-supplementary.pdf]

Supplementary figures

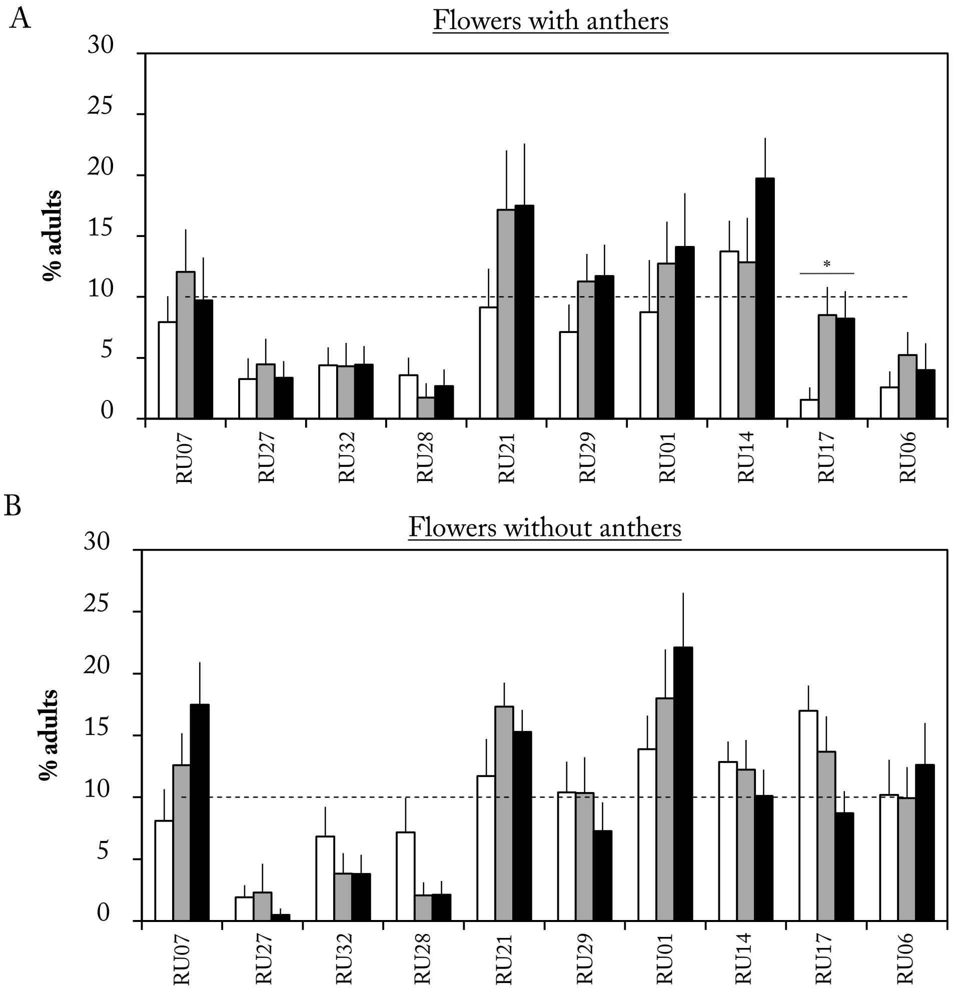

Figure S1. *Frankliniella occidentalis* full choice behavior assay on flowers with and without anthers after 10 minutes (white bars), 1h (grey bars) and 2h (black bars) of 10 *Capsicum* accessions. (A) Mean (±SE) percentage of adult thrips present on flowers with anthers (n = 10 per accession). (B) Mean (±SE) percentage of adult thrips present on flowers without anthers (n = 10 per accession). An asterisk indicates a significant difference within an accession among time points ($P < 0.05$, Friedman ANOVA).

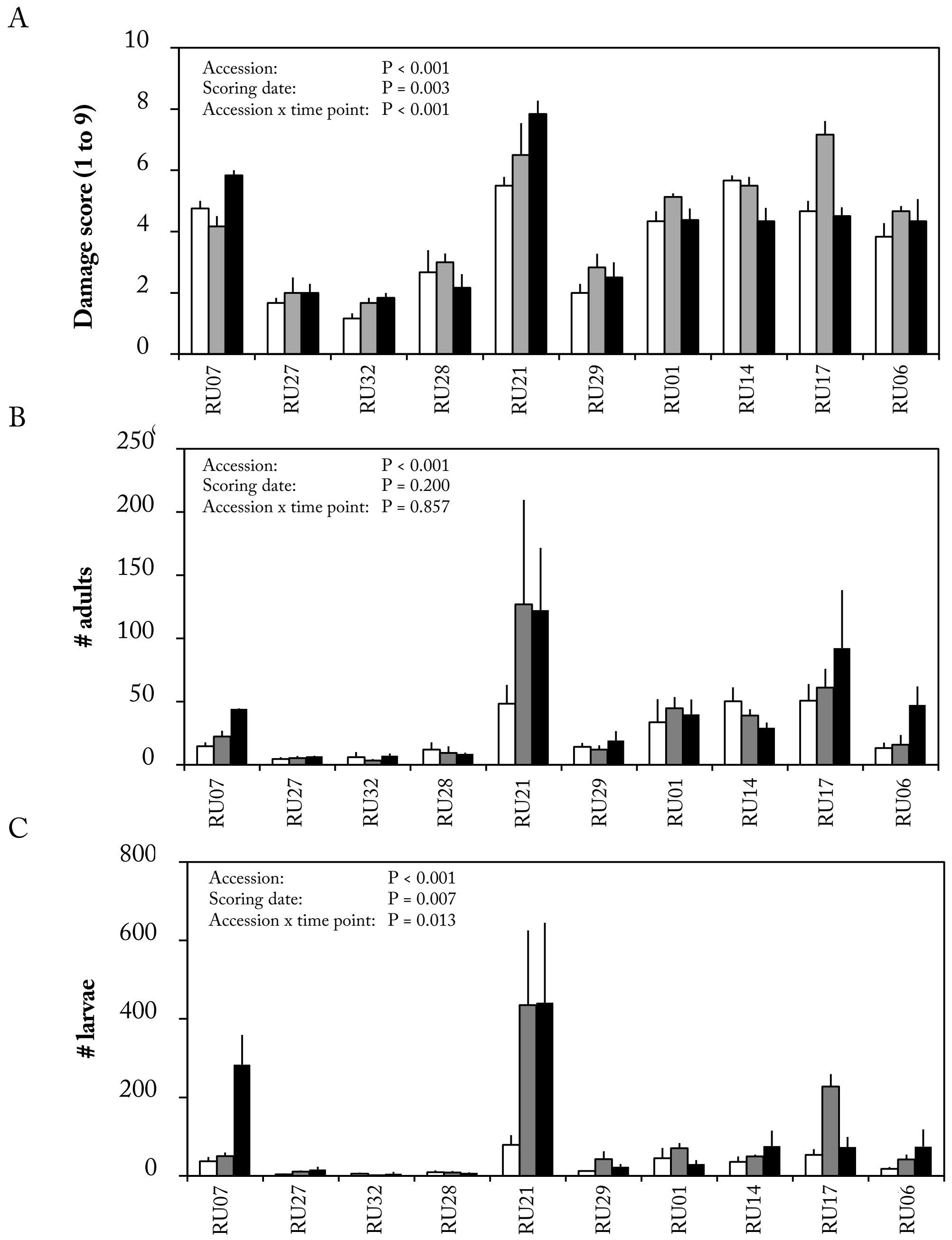

Figure S2. *Frankliniella occidentalis* population development on 10 *Capsicum* accessions 2 (white bars), 3 (grey bars) and 5 weeks (black bars) after inoculation. (A) Mean (±SE) damage scores of whole plant screening (1 little damage, 9 severe damage, n = 3-4 per accession). (B and C) Mean (±SE) number of adults and larvae, respectively (n =3-4 per accession). P-values of overall main effects of accession and scoring data on thrips parameters (ANOVA for independent data), are given in each graph.
